# Caspase-2-Dependent Dendritic Cell Death, Maturation, and Priming of T Cells in Response to *Brucella abortus* Infection

**DOI:** 10.1371/journal.pone.0043512

**Published:** 2012-08-22

**Authors:** Xinna Li, Yongqun He

**Affiliations:** Unit for Laboratory Animal Medicine and Department of Microbiology and Immunology, University of Michigan Medical School, Ann Arbor, Michigan, United States of America; National Council of Sciences (CONICET), Argentina

## Abstract

Smooth virulent *Brucella abortus* strain 2308 (S2308) causes zoonotic brucellosis in cattle and humans. Rough *B. abortus* strain RB51, derived from S2308, is a live attenuated cattle vaccine strain licensed in the USA and many other countries. Our previous report indicated that RB51, but not S2308, induces a caspase-2-dependent apoptotic and necrotic macrophage cell death. Dendritic cells (DCs) are professional antigen presenting cells critical for bridging innate and adaptive immune responses. In contrast to *Brucella*-infected macrophages, here we report that S2308 induced higher levels of apoptotic and necrotic cell death in wild type bone marrow-derived DCs (WT BMDCs) than RB51. The RB51 and S2308-induced BMDC cell death was regulated by caspase-2, indicated by the minimal cell death in RB51 and S2308-infected BMDCs isolated from caspase-2 knockout mice (Casp2KO BMDCs). More S2308 bacteria were taken up by Casp2KO BMDCs than wild type BMDCs. Higher levels of S2308 and RB51 cells were found in infected Casp2KO BMDCs compared to infected WT BMDCs at different time points. RB51-infected wild type BMDCs were mature and activated as shown by significantly up-regulated expression of CD40, CD80, CD86, MHC-I, and MHC-II. RB51 induced the production of cytokines TNF-α, IL-6, IFN-γ and IL12/IL23p40 in infected BMDCs. RB51-infected WT BMDCs also stimulated the proliferation of CD4^+^ and CD8^+^ T cells compared to uninfected WT BMDCs. However, the maturation, activation, and cytokine secretion are significantly impaired in Casp2KO BMDCs infected with RB51 or *Salmonella* (control). S2308-infected WT and Casp2KO BMDCs were not activated and could not induce cytokine production. These results demonstrated that virulent smooth strain S2308 induced more apoptotic and necrotic dendritic cell death than live attenuated rough vaccine strain RB51; however, RB51, but not its parent strain S2308, induced caspase-2-mediated DC maturation, cytokine production, antigen presentation, and T cell priming.

## Introduction

*Brucella* is a facultative intracellular α2-proteobacterium that causes brucellosis, one of the most common zoonotic diseases, in humans and a wide variety of animals [1]. *B. melitensis*, *B. suis*, and *B. abortus* are classified as priority category B pathogens that have the potential for use as a bioterrorism weapon. Infections with *Brucella* occur in humans through inhalation of aerosols, wounds, or consumption of infected food. *B. abortus* causes abortion in cattle and undulant fever in humans. *Brucella* pathogenicity resides mainly in its ability to survive and replicate intracellularly in mononuclear phagocytes and to control host immune responses. After entering the host, *Brucella* is taken up by macrophages and dendritic cells (DCs) [2]. Virulent smooth *Brucella* survive and replicate within these professional phagocytes. The infected host cells play a crucial role in the dissemination of the bacteria in specific locations of the body. Smooth *Brucella* strains contain intact lipopolysaccharide (LPS) O-antigen (*e.g.*, S2308). Rough *Brucella* strains lack the O-antigen. While smooth *Brucella* strains prevent macrophage cell death, rough attenuated *Brucella* strains cannot survive inside macrophages and often induce programmed macrophage cell death [3], [4], [5], [6]. At present, there is no licensed human *Brucella* vaccine. Rough *B. abortus* strain RB51, derived from virulent smooth strain 2308 (S2308), is a live attenuated cattle vaccine strain licensed in the USA and many other countries [7]. *Brucella*-specific T helper type 1 (Th1) response and cytotoxic T lymphocyte (CTL) activities are critical for RB51-induced protective immunity [8].

Dendritic cells (DCs) are the most potent professional antigen presenting cells (APCs) and are critical for bridging innate and adaptive immune responses [9]. DCs capture and process invading microbes and present their antigenic determinants to corresponding lymphocytes. Following antigen uptake in peripheral tissues, immature DCs are converted to a mature and activated phenotype. This process is characterized by up-regulation of different cell surface molecules including CD40, CD80 (also known as B7.1), CD86 (also known as B7.2), and MHC class I and II molecules [10]. Inflammatory stimuli such as TLR ligands or cytokines (*e.g.* TNF-α) promote the maturation process. After mature DCs migrate to a draining lymph node, co-stimulatory molecules such as CD80 and CD86 bind CD28 on naïve T cells, leading to T cell activation. Mature DCs also secrete cytokines, such as IL-12 and IFN-γ. These provide additional signals necessary for the acquisition of the CD8+ T cell effector function. After the primed effector T cells exit the lymph nodes, they recognize and eliminate specific target cells in the cell periphery [11], [12], [13].

Many studies on DC-*Brucella* interactions have been reported in the past decade [14], [15], [16], [17], [18], [19]. *Brucella* has been found to be one of the few bacterial pathogens that can infect and multiply inside DC cells. Billard *et al.* presented direct evidence for a great susceptibility of human monocyte-derived DCs to infections of smooth virulent *B. suis*, *B. abortus*, and *B. melitensis* [14]. Virulent *Brucella* species efficiently invade human monocyte-derived DCs, grow extensively within them and prevent human DC maturation and antigen presentation. DCs infected with wild type virulent *Brucella* do not produce TNF-α. Consequently they exhibit severe maturation impairment, at the phenotypic and at the functional level. They neither secrete IL-12 nor stimulate naïve T-lymphocyte proliferation [15]. The inability of DC maturation was also reported in other reports [18], [20]. However, Zwerdling et al. demonstrated that virulent smooth *B. abortus* strain was able to induce maturation of human DCs [17]. Macedo et al. found that heat-killed *Brucella* was also able to induce muration of murine DCs [19]. The differences of possible virulent *Brucella*-induced DC maturation between these studies might be associated with different cell isolation methods, the concentration of DCs, and the use of different *Brucella* strains [17]. Rough attenuated *B. abortus* strain 45/20 and *B. suis manB* mutant were found to induce strong DC maturation and Th1 responses [15]. While heat-killed, γ-irradiated, and live rough strain RB51 induced higher levels of DC activation compared to smooth virulent strain S2308, only live RB51-infected DCs induce significant TNF-α and IL-12 secretion [16]. However, whether live RB51-infected DCs could prime T cell activation has not been reported. Several *Brucella* proteins, including lipoprotein Omp19 [17], *Brucella* lumazine synthase (BLS) [21], Btp1 [18], and Omp16 [22] have been found to mediate DC maturation. *B. abortus*-exposed MyD88 knockout DCs showed a significant impairment of maturation, shown by an observed decrease in DC expression of CD40, CD86, MHC class II, IL-12 and TNF-α [19].

Caspase-2 is the most conserved caspase across mammal species. Of the family of 13 caspases in mammals, caspase-2 is evolutionally the most homologous to Ced-3, the single caspase that mediates killing and cell death activity in *C. elegans* [23], [24]. Although caspase-2 was found to be essential inducer of apoptosis in oocyte and neuron developments, caspase-2 knockout mice develop normally and are devoid of severe phenotypic abnormalities [25]. The finding suggests that the function of caspase-2 is largely redundant for cellular homeostasis during the early stages of development and in adult tissues as well. However, caspase-2 has been found critical to many biological processes including DNA damage repair, tumor prevention, immune responses to pathogen infections, and aging controls [24]. Krumschnable proposed that caspase-2 may represent the archetype member of the caspase family that combines many different functions in a single enzyme [24]. Caspase-2 activation has been observed in macrophages infected with *Salmonella* [26] and Epstein-Barr virus [27]. Recent studies in our laboratory indicate that vaccine strain RB51, but not its virulent parent strain 2308, induces a caspase-2-dependent apoptotic and necrotic macrophage cell death [3]. Our study also found that caspase-2 mediates a pro-inflammatory cell death in macrophages infected with rough attenuated *B. suis* strain VTRS1 but not its parent virulent strain 1330 [6]. Whether caspase-2 regulates cell death of DCs infected with any pathogens is unknown. It also remains to be explored whether caspase-2 regulates protective T cell immunity against brucellosis and other infectious diseases.

In this study, we report that both live attenuated vaccine strain RB51 and its parent virulent strain S2308 induced caspase-2-dependent apoptotic and necrotic BMDC cell death, and more cell death was observed in S2308-infected wild type BMDCs. We hypothesized that caspase-2 is required to mediate cytokine production, maturation and activation, and antigen presentation of DCs after infection with RB51, leading to *Brucella*-specific T cell priming. This hypothesis is verified experimentally in this study.

## Results

### S2308 and RB51 Induce Caspase-2-regulated Apoptotic and Necrotic BMDCs Cell Death

The programmed cell death of *Brucella*-infected wild type (WT) BMDCs was analyzed by Annexin V (green) and propidium iodide (PI, red) staining. Fluorescein-conjugated Annexin V was used to detect translocation of phosphatidylserine from the inner cell membrane to the outer cell membrane of cells during the early stage of apoptosis. PI stains the DNA of necrotic cells and/or cells at the late stage of apoptosis [28]. The level of cell death significantly increased with increased multiplicities of infection (MOIs) and time of infection (Table 1). Our PI/Annexin V staining analysis indicates that both RB51 and S2308 induced almost 90∼100% apoptotic or necrotic cell death of infected WT BMDCs with a MOI of 50 or 100 at 24 h post infection (Table 1). With a MOI of 20, both RB51 and S2308 induced almost 70∼80% apoptotic or necrotic cell death of infected WT BMDCs at 24 h post infection. The different levels of RB51 and S2308-induced BMDC cell death was observed with a MOI of 20 for 4 h or a MOI of 5 for 24 h. In both cases, more cell death was observed in S2308-infected BMDCs compared to RB51-infected BMDCs (Table 1).

**Table 1:** Kinetic analysis of *Brucella*-induced dendritic cell death.

MOI	H.P.I. a	RB51^b^	S2308^b^
**100**	4	++++	++++
	24	++++	++++
**50**	4	+++	++++
	24	++++	++++
**20**	4	++	+++
	24	+++	+++
**5**	4	+	+
	24	+	++

a Hours Post Infection.

b Results of simultaneous Annexin V and PI staining of dendritic cells infected with indicated bacteria: ++++, 75–100% positive; +++, 50–75% positive; ++, 25–50% positive; +, 5–20% positive; ±, <5% positive; −, no positive staining. A staining positive cell means it is positive in Annexin V staining, PI staining, or staining with both dyes.

**Figure 1:**
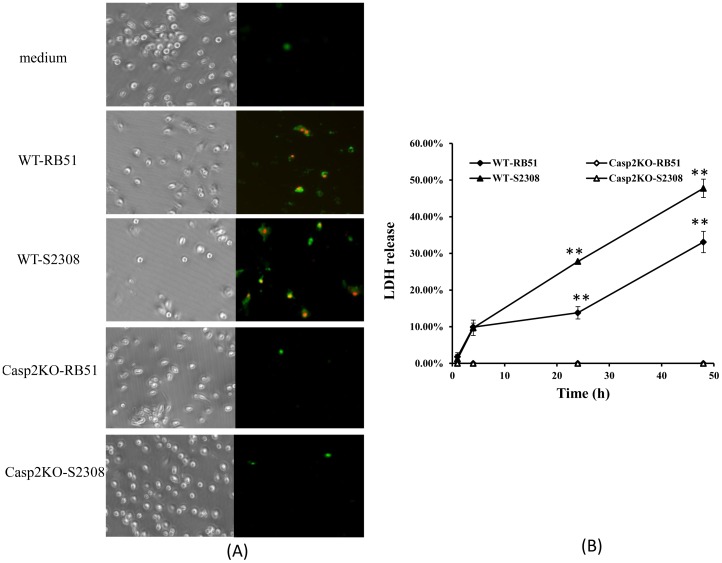
Cell death of BMDCs infected with *B. abortus* strain RB51 and its parent wild type strain S2308. (A) PI and Annexin V staining of *Brucella*-infected BMDCs at 24 h post infection. (B) The LDH release from RB51- or S2308-infected BMDCs derived from WT and Casp2KO mice. The MOI used was 5. The data represent the means ± standard deviations (SDs) from three independent experiments. The asterisk sign (**) represent the significant differences P-value <0.01, respectively, of a LDH release level from infected Casp2KO BMDCs compared to that from WT BMDCs.

Following the protocols of many previous reports [17], [20], our experiments typically used the MOI of 5 for analysis of the interaction between BMDCs and *Brucella* infections. At a MOI of 5, RB51 and S2308 induced both early apoptotic (stained green) and late apoptosis and necrotic (stained green and red) cell death of infected WT BMDCs (Figure 1A). The cytopathic effect of *B. abortus* on BMDCs was further confirmed by a lactate dehydrogenase (LDH) release assay (Figure 1B). LDH is a stable cytosolic enzyme that is released into the extracellular culture medium following loss of membrane integrity resulting from apoptosis or necrosis. The LDH release assay is a nonradioactive cytotoxicity assay used to monitor LDH release from BMDCs with damaged membranes. The total amount of released LDH is the average result of the LDH leak per cell calculated from the number of cells with compromised cell membranes. Therefore, the same amount of LDH might be released by a few cells with severe membrane damage or by a larger number of cells with mild damage. At 24 h and 48 h post infection with RB51 or S2308 with a MOI of 5, we observed that the average level of cytoplasmic LDH released into the S2308-infected BMDCs media was approximately 2-fold elevated compared with that released from RB51-infected BMDCs (Figure 1B). Much higher level of LDH release from RB51 or S2308-infected WT BMDCs was observed at 48 post infection (Figure 1B). No cell death or LDH release was induced in Casp2KO BMDCs with either RB51 or S2308 (Figure 1A and 1B).

**Figure 2:**
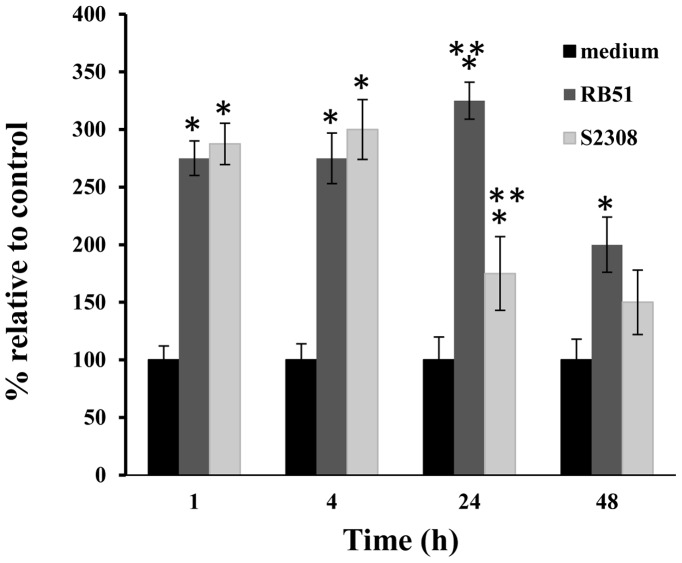
Caspase-2 enzyme activity in infected WT BMDCs induced by RB51 and S2308. The results were quantified by densitometry and normalized to the β-actin content. The data represent the means ± standard deviations from three independent experiments. The sign (*) represents a statistically significant difference (P-value <0.05) of caspase-2 enzyme activity in *Brucella*-infected BMDCs compared to uninfected control. A statistically significant difference of caspase-2 enzyme activities in RB51 or S2308-infected WT BMDCs was labeled with (**) (P-value <0.05).

To further examine whether caspase-2 was activated during *Brucella* infection of WT BMDCs, the caspase-2 enzyme activity was also examined. The caspase-2 enzyme activity in both RB51- and S2308-infected BMDCs was significantly up-regulated at 1 h, 4 h, and 24 h post infection (P-value <0.05). The enzyme activity was reduced at a late infection stage, *i.e.*, 48 h post infection (Figure 2).

### Caspase-2 is Pivotal to Control the Survival and Replication of B. abortus Cells Inside DCs

**Figure 3:**
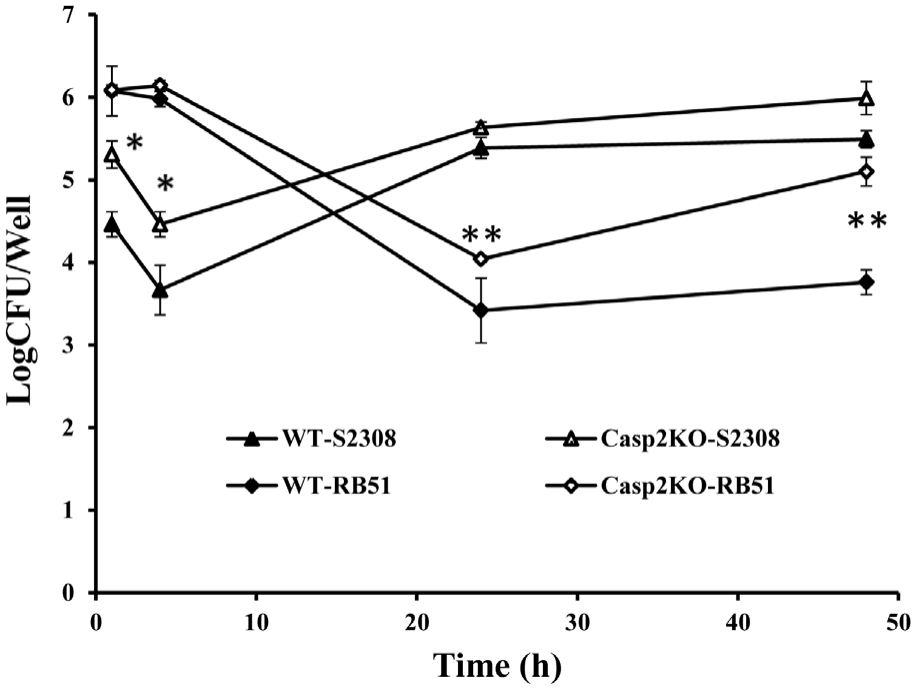
Growth kinetics of RB51 and S2308 inside infected WT and Casp2KO **BMDCs.** WT and Casp2KO BMDCs were infected with RB51 or S2308 at a MOI of 5∶1. The numbers of live intracellular bacteria at different time points post infection were evaluated by CFU examination. Data are means ± standard deviations from three independent experiments. The sign (*) represents a statistically significant difference (P-value <0.05) of S2308 survival inside infected WT and Casp2KO BMDCs. A statistically significant difference of RB51 survival inside infected WT and Casp2KO BMDCs was labeled with (**) (P-value <0.05).

To determine the contribution of caspase-2 to bacterial clearance in DCs, kinetic profiles of intracellular survival of RB51 and S2308 cells in WT or Casp2KO BMDCs were analyzed at several time points post infection (Figure 3). The initial uptake rates of RB51 cells by WT and Casp2KO BMDCs at 1 h post infection were the same (Figure 3). More RB51 bacteria were killed (0.5 log difference) in WT BMDCs compared with RB51 in Casp2KO BMDCs at 24 h post infection. The majority of infected RB51 bacteria were killed during the first 24 h in WT BMDCs, followed by a slight (but not statistically significant) increase of live RB51 cells at 48 h post infection. More RB51 cells in Casp2KO BMDCs were found at 48 h post infection than at 24 h post infection, suggesting survived RB51 began to replicate in Casp2KO BMDCs at the late infection stage. At 24 h and 48 h post infection, Casp2KO BMDCs contained approximately 0.5 log and one log, respectively, higher levels of RB51 bacterial CFUs than WT BMDCs (P-value <0.05).

In contrast to RB51, a lower number of S2308 cells were taken up by WT and Casp2KO BMDCs. At 1 h post infection with S2308, the initial uptake of *Brucella* by Casp2KO BMDCs was one log *Brucella* CFU higher than that for WT BMDCs (Figure 3). Approximately 90% (one log) of S2308 bacteria inside WT or Casp2KO BMDCs were killed during the first 6 h post infection. Afterwards, survived live S2308 bacteria began to replicate in both types of BMDCs (Figure 3). At 24 h post infection, the number of live S2308 cells in Casp2KO BMDCs was higher than that of S2308 cells in Casp2KO BMDCs at 1 h post infection and also higher than the number of RB51 cells found in Casp2KO BMDCs at 24 h and 48 h post infection (Figure 3).

### Caspase-2 is Required for DC Maturation Following Activation with RB51

**Figure 4:**
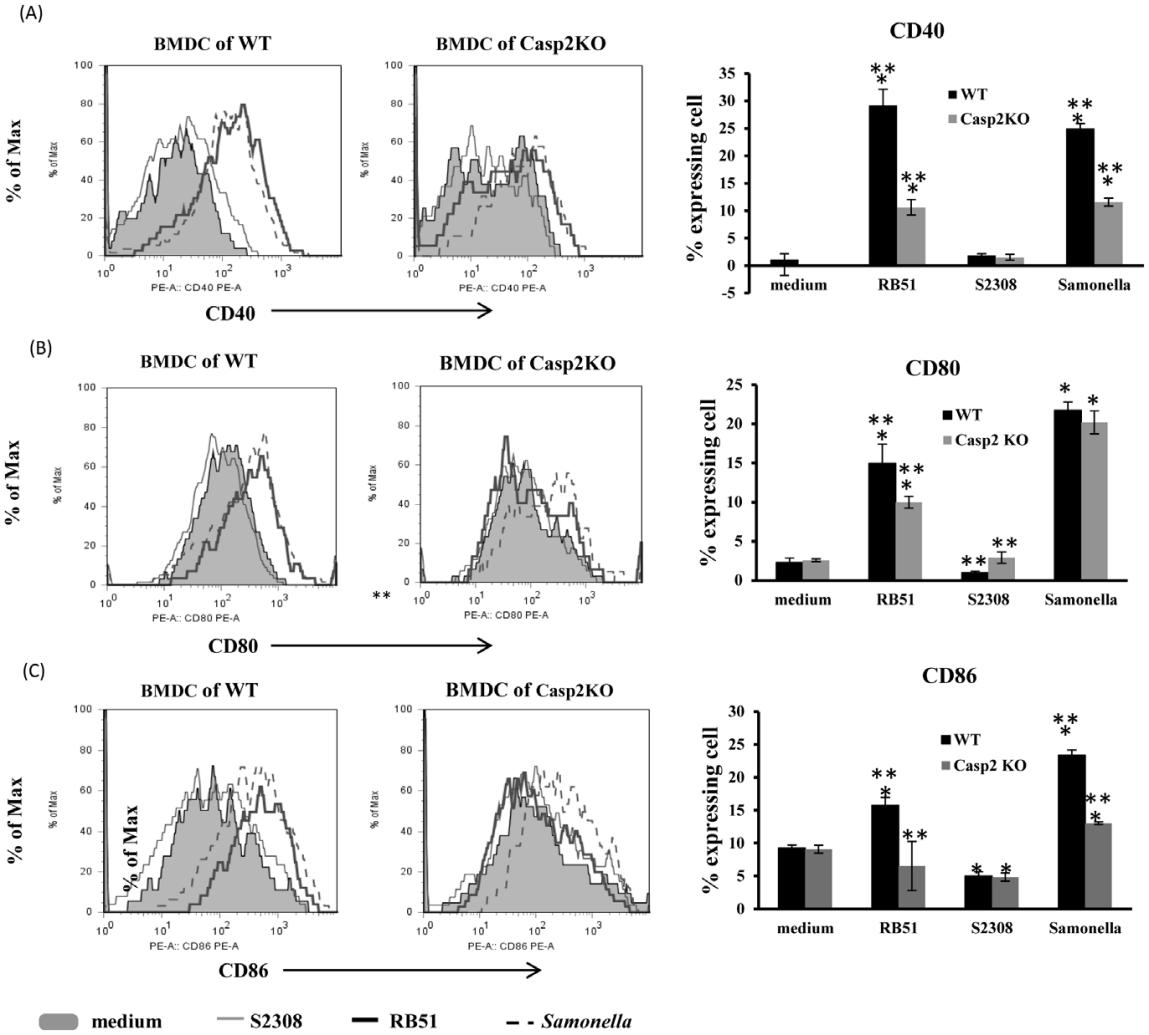
BMDC maturation in response to infection with rough *Brucella* strain RB51 or its parent smooth strain S2308. WT or Casp2KO BMDCs were infected with S2308, RB51, or *S. typhimurium* strain SL1344 (MOI: 5). At 24 h post infection, the expression of three costimulatory cell surface markers CD40 (A), CD80 (B), and CD86 (C) was measured by flow cytometry. For each surface molecule studied, a cytometry analysis histogram from one representative experiment is presented on the left, and the complication histogram on the right includes summarized results (means ± standard deviations of the means) from four independent experiments. The asterisk sign (*) denotes statistically significant difference (P-value <0.05) between an infection group and the medium control group. The sign (**) represents statistically significant difference (P-value <0.05) between WT and Casp2KO BMDCs infected with the same bacteria.

**Figure 5:**
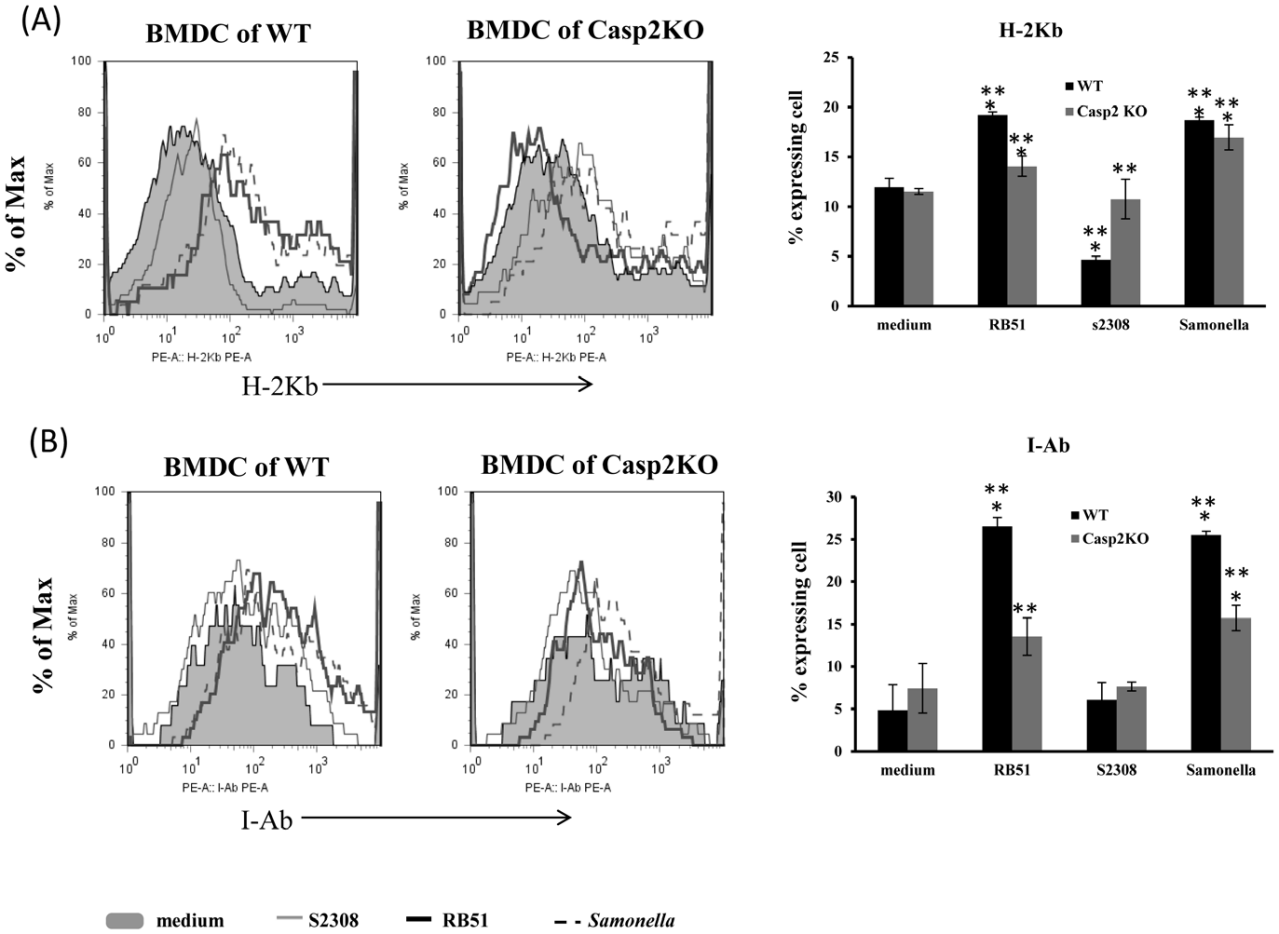
Surface expression of MHC class I and II molecules of BMDCs in response to infection with RB51 or S2308. WT or Casp2KO BMDCs were infected with *Brucella* strains S2308 or RB51, or *S. typhimurium* strain SL1344 (MOI: 5). At 24 h post infection, the surface expression of two MHC class molecules H-2Kb (A) and I-Ab (B) was measured by flow cytometry. For each surface molecule studied, a cytometry analysis histogram from one representative experiment is presented on the left, and the complication histogram on the right includes results (means ± standard deviations of the means) from four independent experiments. Data present means ± SDs of three independent experiments. The asterisk sign (*) denotes statistically significant difference (P-value <0.05) between an infection group and the medium control group. The sign (**) represents statistically significant difference (P-value <0.05) between WT and Casp2KO BMDCs infected with the same bacteria.

To characterize the role of caspase-2 in DC maturation, we stimulated WT or Casp2KO BMDCs with RB51 or S2308 and analyzed the expression of characteristic cell surface markers on the surface of treated BMDCs. RB51-exposed BMDCs matured as shown by enhanced expression of CD40 (Figure 4A), CD80 (Figure 4B), CD86 (Figure 4C), H-2Kb (a mouse MHC I molecule) (Figure 5A), and I-Ab (a mouse MHC II molecule) (Figure 5B) at 24 h after *Brucella* stimulation of immature BMDCs from wild type (WT) mice. Conversely, treatment with S2308 did not induce DC maturation as evidenced by minimal expression of these “costimulatory” and antigen presentation molecules (Figures 4 and 5). These results indicate that RB51 is a strong inducer of DC maturation, but S2308 is not.

RB51-induced BMDC maturation was partially dependent on caspase-2. Specifically, the expression levels of maturation markers including CD40, CD80, CD83, MHC-I, and MHC-II in RB51-infected Casp2KO BMDCs were significantly lower than those in WT BMDCs (P-value <0.05). However, the expression levels of these maturation markers in S2308-infected WT or Casp2KO BMDCs was no greater than that in corresponding BMDCs treated with medium (negative control) (Figures 4 and 5). Furthermore, the expression of CD86 (Figure 4C) and H-2Kb (MHC I molecule) (Figure 5A) in S2308-infected WT or Casp2KO BMDCs was even lower than that in corresponding BMDCs treated with medium (negative control) (P-value <0.05). It is noted that compared to WT BMDCs, *Brucella* strain RB51 or *Salmonalla* strain SL1344-infected Casp2KO BMDCs continued to exhibit a significant increase of CD40, CD80 and MHC-I molecules, and a non-significant increase of MHC-II expression (Figures 4 and 5). Therefore, there was only a partial dependency between caspase-2 and DC maturation.

Since *Salmonella* induces strong maturation of DCs [29], *S. typhimurium* strain SL1344-infected BMDCs was used as a positive control. It was found that SL1344 up-regulated costimulation markers (CD40, CD80, and CD86) (Figure 4) and antigen presentation molecules (MHC classes I and II) (Figure 5). These findings are consistent with previous report [30]. Our results further indicated that compared with wild type BMDCs, *Salmonella*-infected Casp2KO BMDCs produced significantly down-regulated CD40, CD86, and MHC II molecule I-Ab (P-value <0.05) (Figures 4 and 5). However, no significant changes were observed in the other molecules (CD80 and MHC class I molecule H-2Kb).

### Cytokine Production by Brucella-stimulated BMDCs is Caspase-2-dependent

**Figure 6:**
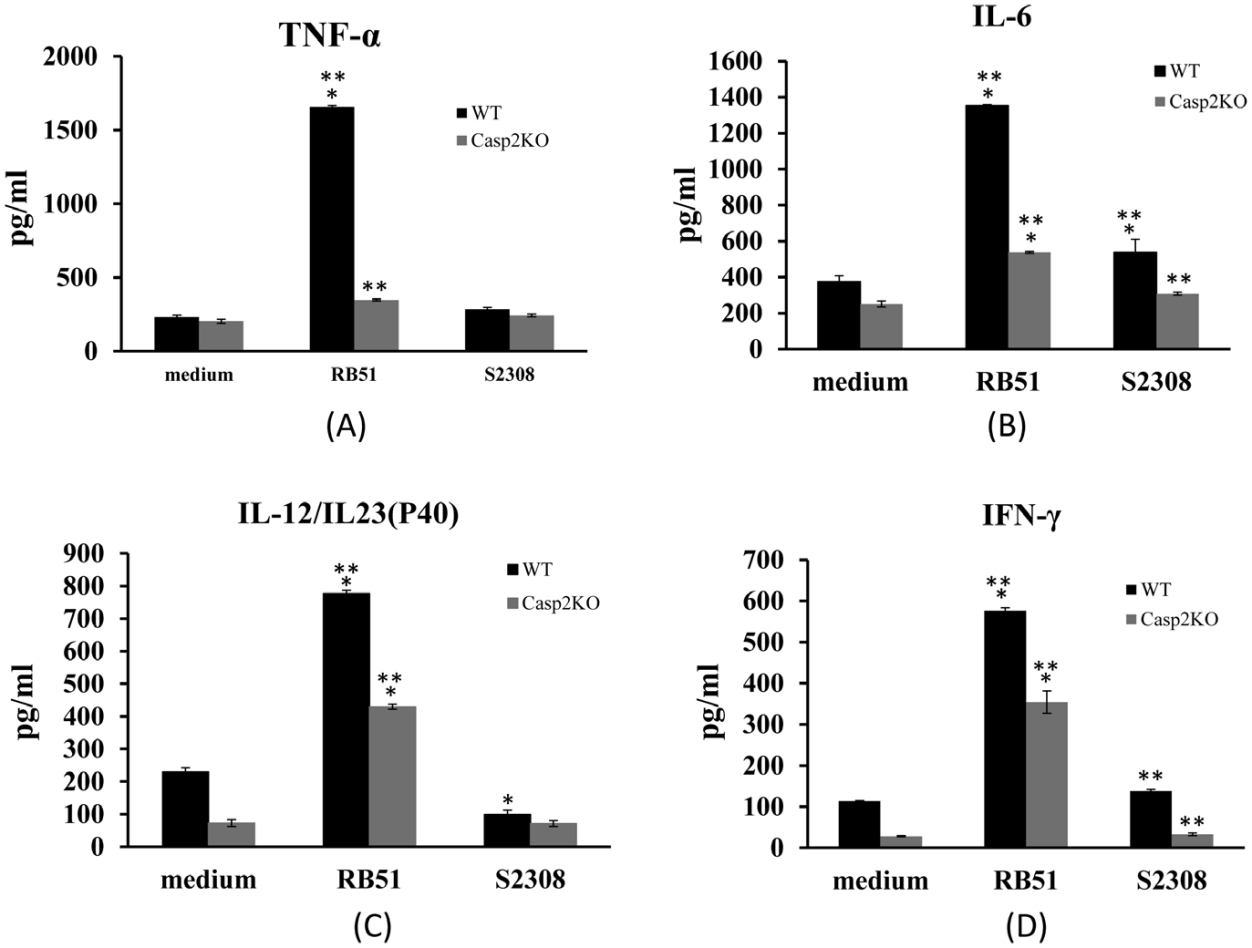
The effects of caspase-2 on production of cytokines in *Brucella*-infected BMDCs. To assess the effect of caspase-2 on DC function, the protein levels of TNF-α (A), IL-6 (B), IL12/IL23p40 (C), and IFN-γ (D) from the culture supernatants of S2308- or RB51-infected WT or Casp2KO BMDCs were collected and analyzed using ELISA. Supernatants were obtained at 24 h post infection. The results represent cytokine secretion (means ± standard deviations of the means of three experiments). The asterisk sign (*) denotes statistically significant difference (P-value <0.05) between an infection group and the medium control group. The sign (**) represents statistically significant difference (P-value <0.05) between WT and Casp2KO BMDCs infected with the same bacteria.

Among the cytokines secreted by DCs upon pathogen phagocytosis, the multipotent inflammatory cytokine TNF-α is required for host defense against a variety of intracellular pathogens and plays a crucial role in DC maturation [31]. The lack of DC maturation during infection in smooth virulent *B. suis* is related to the absence of TNF-α secretion [20]. As expected, with the up-regulation of cell surface markers, RB51 infection of WT BMDCs induces a significantly increased production of TNF-α at 24 h post infection (P-value <0.05) (Figure 6A). TNF-α production by RB51-infected Casp2KO BMDCs was partially abrogated as compared to that in infected WT BMDCs (P-value <0.05). In contrast, compared to uninfected control, an infection with S2308 failed to induce significantly changed secretion of TNF-α in WT or Casp2KO BMDCs (Figure 6A).

IL-6 is a key cytokine in DC development [32]. IL-6 is also an important inflammatory response mediator in DCs [33], [34]. S2308 induced mild but statistically significant change in IL-6 production in WT BMDCs compared to medium control (P-value <0.05) (Figure 6B). However, no statistically significant change of IL-6 production in Casp2KO BMDCs was detected. In contrast, RB51 demonstrated more than two-fold of increased production of IL-6 in WT BMDCs compared to the medium control or the S2308 infection group. The knockout of caspase-2 in BMDCs dramatically decreased the production of IL-6 in RB51-infected Casp2KO BMDCs (Figure 6B).

IL-12 production by DCs is critical for driving a protective CD4 Th1 type immune response and the clearance of intracellular bacteria [2] and triggers the production of IFN-γ, a pivotal cytokine involved in the control of murine brucellosis [35], [36], [37], [38]. When mature DCs present antigens to naïve T cells and stimulate their proliferation, they also induce subsequent polarization of an adaptive immune response towards a Th1 and a Th2 profile by secreting different cytokines. To determine whether caspase-2 is involved in IL-12 and IFN-γ production by DCs, the IL-12/IL-23p40 and IFN-γ concentrations in the culture supernatants of WT and Casp2KO BMDCs infected with RB51 and S2308 were measured. Compared to uninfected BMDCs, approximately four fold of IL-12/IL-23p40 production (Figure 6C) and approximately five fold of IFN-γ production (Figure 6D) were detected in RB51-infected WT and Casp2KO BMDCs. The levels of IL-12 and IFN-γ in the supernatants of S2308 or RB51-infected Casp2KO BMDCs were reduced by approximately 50% compared with those of S2308 or RB51-infected WT BMDCs. It suggested that the IL-12 and IFN-γ productions were partially dependent on caspase-2 and there also exists a caspase-2-independent pathway(s) of cytokine induction. The levels of IL-12 and IFN-γ in the supernatants of S2308-infected WT and Casp2KO BMDCs were almost absent compared with RB51-infected WT and Casp2KO BMDCs.

### Caspase-2 is Required for Naïve T-lymphocyte Proliferation Following Stimulation with RB51-infected BMDCs

**Figure 7:**
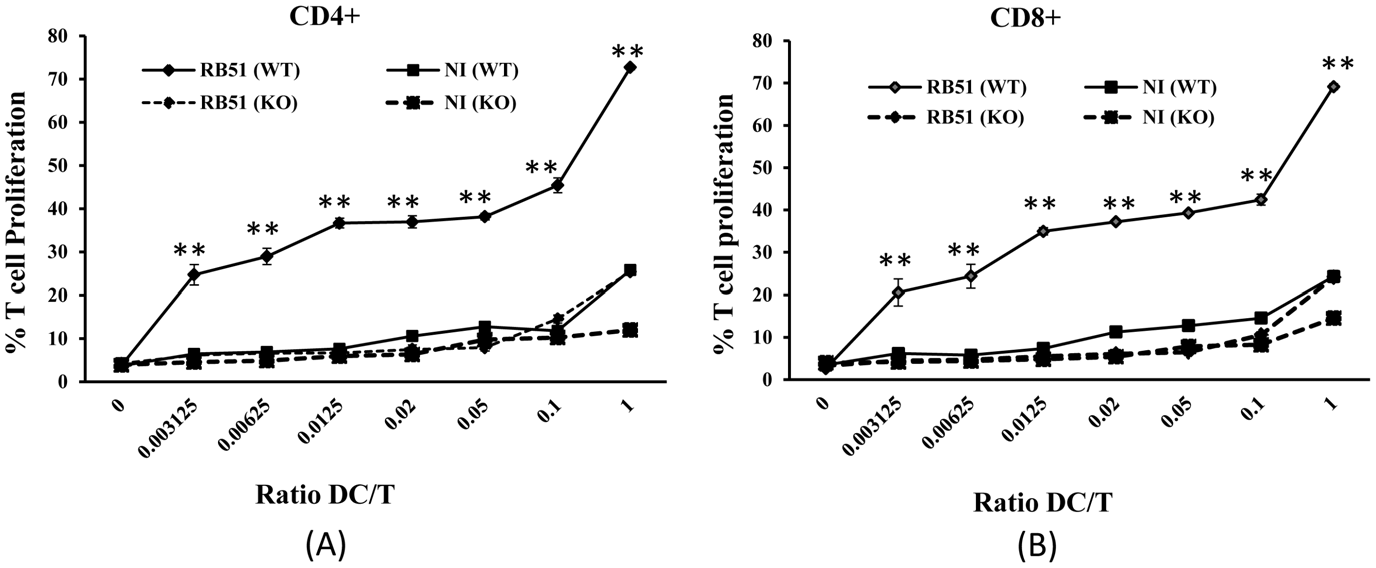
Effect of caspase-2 on priming of allogeneic T-cells through *Brucella*-infected BMDCs. At 24 h post infection, WT and Casp2KO BMDCs that were infected or not infected (NI) with RB51 were co-cultured with CFSE-labeled naïve CD4+ T cells (A) or CD8+ T cells (B) from BALB/c mice for 5 days at different BMDC/T-cell ratios. The proliferation of naïve T-lymphocytes was analyzed by flow cytometry. The data are means ± standard deviations of the means of five independent experiments. Statistical differences for comparisons with uninfected DCs are indicated by the sign (**) (P-value <0.01).

Mature and activated DCs are potent antigen-presenting cells for the priming of naïve T cells [39]. To study the functional effect of the BMDC maturation after *Brucella* infection, we examined whether RB51-induced BMDC maturation could prime allogeneic T cell proliferation. RB51-infected immature WT or Casp2KO BMDCs (from C57BL/6 background) were mixed with naïve BALB/c T cells and stained with carboxyfluorescein diacetate succinimidyl ester (CFSE) using a series of BMDC/T-cell ratios. In such a typical allogeneic assay, the fact that DCs were infected is irrelevant to the recognition by T-cell receptor (TCR) since the T cells were not antigen-specific. However, the infection of DCs modulates the expression of costimulatory molecules on cell surface. In such a context, antigen-treated DCs turn to be efficient to stimulate allogeneic naïve T cells [40]. T-lymphocyte proliferation was determined at five days later by flow cytometry analysis of the decrease in CFSE. WT BMDCs infected with RB51 clearly showed a high capacity to induce the response of naïve CD4^+^ T cells (Figure 7A) and CD8^+^ T cells (Figure 7B) than immature uninfected BMDCs (P-value <0.01). The proliferation level of stimulated CD4^+^ or CD8^+^ T cells was approximately 70% when the DC/T-cell ratio was 1∶1. However, RB51-infected BMDCs from Casp2KO mice were unable to effectively stimulate naïve CD4^+^ or CD8^+^ T cells. At a DC/T-cell ratio of 1∶1, only approximately 20% proliferation of CD4^+^ or CD8^+^ T cells was detected in RB51-infected Casp2KO BMDCs. This level of proliferation was similar to that obtained with uninfected WT BMDCs (Figure 7A and B). Therefore, RB51-induced maturation of WT BMDCs was able to induce T cell proliferation, but RB51-infected Casp2KO BMDCs was not.

## Discussion

In this study, we confirmed that rough attenuated *B. abortus* vaccine strain RB51, but not its parent virulent smooth strain 2308 (S2308), induce DC maturation and T cell priming. We are unique in providing evidences that both S2308 and RB51 induce apoptotic and necrotic DC death and more cell death is induced by S2308. Caspase-2 is required for induction of *Brucella*-induced DC cell death. The knockout of caspase-2 leads to enhanced uptake of smooth strain S2308 (but not RB51) and facilitates survival of both S2308 and RB51 inside BMDCs. Our studies also indicate that caspase-2 is required for RB51-induced DC maturation and T cell priming. Caspase-2 also up-regulates the productions of several cytokines including TNF-α, IL-6, IL- 12/IL23p40, and IFN-γ, which stimulate DC maturation and T cell activation. In contrast, S2308 does not induce wild type or Casp2KO BMDC maturation and priming of T cells.

In contrast to the macrophage cell death induced by RB51 but inhibited by S2308 [3], both S2308 and RB51 induced apoptotic and necrotic cell death of infected wild type BMDCs (Table 1 and Figure 1). Furthermore, S2308 induced more apoptotic and necrotic cell death of wild type BMDCs than RB51 (Figure 1). The opposite phenotypes in macrophages and DCs treated with virulent S2308 or attenuated RB51 are likely associated with the different roles of these two host cell types and how virulent *Brucella* becomes a stealthy pathogen [41]. Macrophages are specialized phagocytic cells that attack foreign bacterial pathogens. The prevention of macrophage cell death by virulent *Brucella* supports the survival and replication of virulent *Brucella* inside macrophages. In contrast, the induction of programmed cell death of macrophages infected with rough attenuated vaccine strain RB51 benefits the host as a whole, by exposing intracellular *Brucella* to more hostile extracellular environment. DCs mainly function as antigen-presenting cells that process antigen material and present it on the surface to other cells of the immune system. The DC cell death induced by S2308 infection results in less chance of DC maturation and its presentation of S2308 antigens to T cells. In this sense, the induction of DC cell death supports *Brucella* pathogenesis. Meanwhile, the DC cell death benefits the host by preventing extensive *Brucella* spreading (see below). In contrast, less DC cell death is induced by attenuated RB51, which benefits the host through more efficient RB51 antigen presentation. Compared to macrophages, DCs are often more efficient at undergoing pathogen-induced apoptosis as a mechanism to limit intracellular replication and pathogen dissemination [42]. Similar phenomenon was also observed in *Yersinia enterocolitica* [43]. Wild type virulent *Yersinia enterocolitica* induces apoptosis of infected DCs [43]. However, an attenuated YopP-deficient *Yersinia* mutant does not induce DC cell death. Infection of virulent *Legionella pneumophila* also induces cell death in DCs [42].

Two interesting phenomena appear to co-exist: (i) DCs may constitute a replication niche for *B. abortus*, and (ii) *Brucella* infection can induce DC cell death and RB51 infection can even activate DCs. These two phenomena need to achieve a final balance. Virulent *Brucella* uses DCs as a replication niche to avoid the host cytokine function, survive, and replicate. After replication, virulent *Brucella* can induce the DC cell death and spread out of DCs. In the context of *Brucella* infection of DCs, DCs function as a “Trojan Horse” to systemically disseminate *Brucella* internalized at peripheral sites of infection [18], [44]. Virulent *Brucella* somehow manages the balance of keeping infected DCs alive for bacterial replication and killing the infected DCs for bacterial dissemination. Meanwhile, the cell death does not induce antigen presentation. DCs undergoing apoptosis are not efficient antigen presenting cells (APCs) in vivo. A decrease in DC numbers at the location of an infection may impair the nascent T cell-dependent response. When RB51-infected DCs remain live, the infected DCs may spread systematically, get activated, and prime T cells at different lymphatic locations. When RB51-infected DCs die, the released *Brucella* antigens may activate neighboring DCs. The host appears to control the vaccine strain well and maintain a balance of DC activation and cell death after RB51 infection. This may form the basis of vaccine RB51-induced adaptive immunity against future virulent *Brucella* infection.

Our results demonstrate that caspase-2 mediates apoptotic and necrotic cell death in DCs infected with *Brucella* strain RB51 or S2308. Minimal cell death in RB51 or S2308-infected BMDCs from Casp2KO was observed (Figure 1). This phenomenon is similar to that observed in rough (including RB51, RA1, and VTRS1) or smooth *Brucella* (including S2308 and S1330)-infected macrophages that were pre-treated with a caspase-2 inhibitor [3] or bone-marrow derived macrophages from Casp2KO mice [6]. The exact mechanism by which caspase-2 regulates programmed cell death of infected DCs remains unknown. Structurally, caspase-2 resembles an initiator caspase, with a caspase activation and recruitment domain (CARD) for auto-activation and binding to other molecules. However, caspase-2 also has a cleavage site, and its *in vitro* cleavage activity resembles that of the death effectors, caspase-3 and -7. Furthermore, caspase-2-induced cell death may be independent or dependent on the mitochondrial cell death pathway [24], [45]. These factors make it difficult to classify caspase-2. Our previous studies indicate that the activation of caspase-2 in *Brucella*-infected macrophages increases mitochondrial membrane permeability and results in cytochrome *c* release [3]. Furthermore, such a caspase-2-mediated cell death is proinflammatory and regulated by NF-κB [6]. It remains to be determined whether the caspase-2 regulatory mechanism found in infected macrophages also occurs in *Brucella*-infected DCs. On the other hand, apoptotic pathways involving caspase-3, caspase-8 and caspase-9, which are intact in Casp2KO mice, are well characterized. The complete abrogation of *Brucella*-induced cell death in BMDCs from the Casp2KO mice (Figure 1) is intriguing. It is likely that as an initiator caspase, caspase-2 functions as a master regulator of several specific cell death pathways involving caspase-3, caspase-8 and caspase-9 in dendritic cells. The unpublished preliminary data recently obtained in our laboratory indicates that caspase-2 plays such a master regulatory role in *Brucella*-infected macrophages. Further analysis of this possible mechanism in dendritic cells is under investigation.

Our results suggest that caspase-2 regulates the uptake, survival, and replication of *Brucella* inside DCs (Figure 3). Interestingly, caspase-2 appears to play a different role in regulating the uptake of smooth and rough *Brucella* (Figure 3). Similar amounts of RB51 cells were taken up by WT and Casp2KO BMDCs. Compared to RB51, a less amount of S2308 cells were taken up by WT and Casp2KO BMDCs at the early infection stage. Compared to WT BMDCs, Casp2KO BMDCs phagocytozed significantly more S2308 cells (but not RB51). It is likely that caspase-2 regulates some gene(s) involving the process of DC phagocytosis of wild type smooth strain S2308. The difference in phagocytosis of smooth and rough *Brucella* in WT BM DCs is similar to the phenomenon in *B. abortus* infected WT macrophages where more rough *Brucella* cells are taken up by macrophages than smooth *Brucella* [3], [5], [46]. While smooth brucellae invade macrophages through lipid-rafts, rough brucellae do not [47]. Such a mechanism may also apply to DCs. It is likely that caspase-2 plays an inhibitory role in lipid raft-mediated uptake of smooth *Brucella* in DCs. Furthermore, our study identified increased numbers of S2308 and RB51 in Casp2KO BMDCs compared to WT BMDCs (Figure 3). The increased brucellae in Casp2KO BMDCs might be due to the inhibition of programmed cell death of infected Casp2KO BMDCs (Figure 1). However, it is also likely that caspase-2 regulates other innate bactericidal activities that are not directly related to cell death pathways.

Caspase-2 is critical for DC maturation after *Brucella* infection. Vaccine strain RB51 is able to induce maturation and activation of infected BMDCs derived from wild type mice (Figure 4 & 5). These results are consistent with previous reports [16], [18]. It is our new finding that the majority of infected RB51 bacteria were killed during the first 24 h in wild type DCs, followed by a slight increase of live RB51 cells at 48 h post infection (Figure 3), and RB51 induces caspase-2-mediated DC maturation and cytokine production (Figures 4 and 5). It is possible that there is a special type of interaction between RB51 and DCs, and such an interaction induces the caspase-2-mediated DC maturation and cytokine production. The costimulatory molecules (CD40, CD80, and CD86) participate in T-cell/DC interaction within the lymph node and are crucial for T–cell activation. These costimulatory molecules in RB51-infected Casp2KO BMDCs were not stimulated, suggesting that caspase-2 regulates the pathways towards the stimulation of the costimulatory molecules. To stimulate antigen-specific T cells, peptidic antigens derived from the degradation of pathogen proteins are loaded onto major histocompatibility complex class II (MHC-II) and MHC-I to be recognized by CD4^+^ and CD8^+^ T cells, respectively. Infection of WT BMDCs with RB51 results in strong expression of these two antigen-presenting molecules. This is in agreement with our previous report that RB51-stimulated CD4^+^ and CD8^+^ T cells play synergistic role in attacking S2308-infected target cells [8]. The protective immunity induced by RB51 is exclusively provided by a T-cell response because passive transfer of serum from RB51-vaccinated mice did not provide protection against mouse infection with live virulent strain S2308 [48]. The fact that RB51 could not stimulate the MHC-I and MHC-II molecule expression at the surface of Casp2KO BMDCs suggests that caspase-2 is required for induction of protective *Brucella* immunity. S2308 could not induce DC maturation and T cell priming in WT BMDCs, consistent with previous report that live smooth virulent *Brucella* did not prime T cells [15]. It is interesting that incapability of inducing DC maturation by S2308 parallels with the induction of BMDC apoptosis by S2308 (Figure 1). It has been shown that DC maturation and antigen presentation is regulated by TNF-α [20]. The production of TNF-α was inhibited at 24 h post S2308 infection (Figure 6). Therefore, the S2308-induced DC cell death is mediated by caspase-2 and is unlikely to be mediated by TNF-α. However, it is possible that TNF-α production in DCs after RB51 infection plays a regulatory role in DC cell death. It is indeed found by our previous study that TNF-α partially mediates the cell death of macrophages infected with live attenuated rough strain VTRS1 [6]. The knockout of caspase-2 does not change the results of DC maturation and TNF-α production in S2308-infected BMDCs. However, the TNF-α production in VTRS1-infected macrophages appeared to be regulated by caspase-2 [6]. Therefore, in host cells infected with virulent smooth or attenuated rough *Brucella* strains, TNF-α appears to be regulated differently, and TNF-α may play different roles in the induction of host cell death. In our study, *Samonella* was used as a control for DC maturation analysis. Our results indicated that caspase-2 also mediates DC maturation in *Samonella*-infected BMDCs (Figures 4 and 5). This suggests that the caspase-2-mediated DC maturation is a generic phenomenon and may occur in other pathogen-infected DCs.

To address how caspase-2 regulates T cell priming through DC antigen processing, we compared the production of many cytokines in WT and Casp2KO mice. TNF-α participates in DC maturation and critical to control murine brucellosis [20], [49]. IFN-γ and IL-12 can be produced by DCs, enhance the antigen presenting capabilities of DCs, and support initiation and modulation of adaptive immune responses [50]. Higher IL-6 levels were correlated with DC content and development [32]. In contrast to S2308, RB51 was able to trigger a potent secretion of TNF-α, IL-12/IL23p40, IFN-γ, and IL-6 from WT BMDCs. However, the production levels of these proinflammatory cytokines are dramatically lower in Casp2KO BMDCs compared with WT BMDCs (Figure 6). It is suggested that caspase-2 participates in stimulating the secretion of cytokine (TNF-α, IL-12/IL23p40, IFN-γ and IL-6) in RB51-infected DCs.

How exactly rough and smooth *Brucella* strains interact with caspase-2 in DCs is unclear. In macrophages, it is widely accepted that *Brucella* LPS O-antigen, which exists in smooth strains but does not exist in rough strain, functions as a barrier that prevents *Brucella* proteins from interacting with macrophage proteins including caspase-2 [3], [51]. This explanation does not apply to DCs since in contrast to macrophages, S2308-infected DCs undergo rapid cell death. It is likely that O antigen at the surface of smooth *Brucella* strains does not have any or only has limited effect on the induction of DC maturation. Several *Brucella* proteins, including lipoprotein Omp19 [17], *Brucella* lumazine synthase (BLS) [21], Omp25, Omp16 [22] and BvrR, have been found to mediate DC maturations [20], [52]. These *Brucella* Proteins may interact with DCs via an O antigen-independent pathway. A specific interaction between RB51 and DC molecules may exist, and such an interaction causes the maturation of caspase-2-mediated DC maturation and cytokine production. The specific pathways of S2308-induced DC cell death and RB51-induced DC maturation deserve further investigations.

No studies have been reported in terms of the role of caspase-2 in DC maturation and T cell priming. For the first time, our results indicate that caspase-2 is required for DC priming of T cells (Figure 7). The study of the intricate interactions between *Brucella* infection and caspase-2-mediated DC maturation and T cell priming is very important for better understanding *Brucella* pathogenesis and host protection *in vivo*. In gut Peyer’s patches, DCs are separated from the intestinal lumen by only a single layer of cells, the follicle-associated epithelium (FAE) [53]. FAE contains antigen-transporting microfold (M) cells and a small number of DCs. The DCs in Peyer’s patches are constantly exposed to foreign antigens including infecting *Brucella* in the gut. Salcedo et al showed that soon after *B. abortus* S2308 inoculation in intestinal loops, dendritic cells from ileal Peyer’s patches become infected [18]. Cattle vaccine strain RB51 is typically administrated subcutaneously in cattle and intraperitoneally in an established mouse model. After administration, the vaccine strain will be able to interact with the DCs locally and systematically, leading to DC maturation and T cell priming. The dissection of the caspase-2-mediated DC immune network against *Brucella* infection would help the rational design of vaccines against *Brucella* and other infectious intracellular pathogens.

## Materials and Methods

### Mice

#### Ethics statement

Animal work in this study was carried out in strict accordance with the recommendations in the Guide for the Care and Use of Laboratory Animals of the USA National Institutes of Health. Mice were used under animal care protocol (#09695) approved by the Committee on Use and Care of Animals (UCUCA) of the University of Michigan, Ann Arbor, MI, USA. The specific study reported in this manuscript has also been approved by the UCUCA review board or ethics committee in the University of Michigan.

Female 6–8 week-old C57BL/6 mice and BALB/c were obtained from Jackson Laboratory (Bar Harbor, Maine, USA). The caspase-2 knockout (Casp2KO) mice were originally generated by Junying Yuan and kindly provided by Dr. Brian Herman of the University of Texas Health Science Center at San Antonio with Dr. Yuan’s consent [25]. The deletion inactivates both the long and short form of caspase-2. The mice were backcrossed with C57BL/6 once in in the Unit for Laboratory Animal Medicine at the University of Michigan Medical School, and then used as founders. Casp2KO and wild type (WT) mice with similar ages were applied in the experiments.

### Bacteria

*B. abortus* strains RB51 and S2308, kindly provided by Dr. Gerhardt Schurig at Virginia Tech, were cultured using Tryptic Soy Broth (TSB) or Tryptic Soy Agar (TSA) (Beckton Dickinson,. Cockeysville, MD, USA). All experiments involving live virulent *Brucella* were performed in a Biosafety Level (BSL-3) facility at The University of Michigan Medical School. Wild type *Salmonella typhimurium* strain SL1344 was obtained from Dr. Mary O’Riordan in The University of Michigan Medical School.

### Primary Dendritic Cell Preparation

Bone marrow-derived DCs (BMDCs) were prepared, as previously described [54]. Briefly, tibias and fibulas from 6–8 week-old C57BL/6, or Casp2KO mice, were incised and the bone marrow (BM) cells removed. Following lysis and removal of red blood cells, the cells were resuspended and plated in RPMI 1640 complete media supplemented with 10% heat-inactivated fetal bovine serum and 10 ng/ml rGM-CSF and 10 ng/ml rIL-4 (PeproTech, Rocky Hill, NJ). The cells were cultured at 37°C under 5% CO_2_. Fresh medium containing 10 ng/ml rGM-CSF and 10 ng/ml rIL-4 were added at day 4. The cells harvested on day seven typically contained approximately 70% CD11c^+^ cells and contained low levels of major histocompatibility complex (MHC) class II, low levels of CD40 and CD86 expression. These findings are consistent with immature DCs. Flow cytometry of these cytokines was performed to confirm the activation status of the DCs.

### Infection of BMDCs with Brucella

Unless specified elsewhere, BMDCs were normally harvested and plated at 5×10^5^ cells per well in 24-well plates. Fresh bacteria were grown in TSB at 37°C with vigorous shaking (200 rpm) overnight. BMDCs were infected with live RB51 or S2308 at a multiplicity of infection (MOI) of five or other specified MOIs in culture medium lacking antibiotics. An MOI of five is commonly used for *Brucella* infections of DCs [17], [20]. Infection was enhanced by a short spin at 400×g for five minutes at room temperature. After incubation for 1 h at 37 °C in 5% CO_2_, the infected cells were washed for three times with medium containing 50 µg/mL gentamicin (Sigma, St. Louis, MO) that is able to kill extracellular bacteria. The stimulated cells were incubated for 1 h, 4 h, 24 h, or 48 h in complete medium containing 50 µg/ml gentamicin. At each time point, the cells were harvested and used as described below. Uninfected cells containing only culture medium were used as a negative control. BMDCs treated with *S. typhimurium* strain SL1344 at a MOI of five under the same protocol served as a positive control.

### Determination of Programmed Dendritic Cell Death

BMDCs derived from wild type mice (WT BMDCs) or Casp2KO mice (Casp2KO BMDCs) were infected with RB51 or S2308 at a MOI of five as detailed above. Apoptotic or necrotic BMDCs were detected using two approaches. In the first approach, cells were stained with Annexin V (green dye) and propidium iodide (PI, red dye) using an Annexin V-FLUOS staining kit (Roche Diagnostics Corporation, Indianapolis, Ind.). Briefly, RB51 and S2308-infected WT or Casp2KO BMDCs were incubated with Annexin V and PI at room temperature for 20 min and observed by fluorescence microscopy (Nikon TE2000-S microscope). Images were photographed with an RT Slide Spot digital camera. Apoptotic and necrotic cell numbers were counted in at least three representative fields containing 200 cells per field. In the second method, the cell viability of BMDCs, after infections with *B. abortus,* was measured by lactate dehydrogenase (LDH) release assay [3]. Cells cultured in triplicate in 96-well plates were infected with RB51 and S2308 as described above. At various time points over the course of 48 h, the culture supernatants were collected and the LDH levels measured using Promega Cytotox 96 assay kit according to the manufacturer’s instructions. To reduce the LDH background from fetal bovine serum, the supernatants were diluted 1∶1 with phosphate buffered saline (PBS) prior to the assay. The percent of membrane damaged (dying cells) is expressed as a percentage of maximum LDH release, i.e., 100× (optical density at 490 nm [OD490] of infected cells - OD490 of uninfected cells)/(OD490 of lysed uninfected cells - OD490 of uninfected cells). The data presented represent the average ± standard deviation of at least three separate experiments.

### Assay of Brucella Survival Inside BMDCs

RB51 and S2308 were used to infect WT or Casp2KO BMDCs with a MOI of five. To assess the intracellular survival of *Brucella* BMDCs, the cells were lysed with 1 ml 0.1% (vol/vol) Triton X-100 in sterile water at selected time points (1 h, 4 h, 24 h, and 48 h, respectively). The colony forming units (CFUs) were obtained by plating a series of dilutions on TSA plates [55]. All experiments were conducted in triplicate.

### Caspase-2 Activity Assays

WT BMDCs were cultured in T-25 flasks and infected with RB51 or S2308 with a MOI of five. The caspase-2 enzyme activity was determined by monitoring proteolysis of the appropriate colorimetric substrates using the Caspase Colorimetric Assay kits (Promega, CA). Specifically, samples (50 µl) of the lysate were aliquoted into wells in a 96-well microplate, and 50 µl of reaction buffer containing 10 mM DTT were then added to each well. The caspase-2 substrate VDVAD pentapeptide (5 µl, final concentration 4 mM) was added to the appropriate wells. The plate was then incubated at 37°C for 2 h. Absorbance at 405 nm was read in a microplate reader using a VERSA MAX microplate reader (Molecular Devices). All experiments were conducted in triplicate. The absorbance from treated samples was compared with the corresponding untreated control to allow the determination of the change in caspase-2 activity.

### Phenotypical Analysis by Flow Cytometry

BMDCs (1×10^6^ cells in one ml per well in 24-well plates) were infected with *Brucella* at the MOI of 5. At 24 h post infection, BMDCs were labeled individually with rat anti-mouse monoclonal antibodies (mAbs) against a number of compounds including CD40, CD80, CD86, CD11c, CD11b, H-2Kb, and I-Ab (BD Pharmingen, San Diego). Flow cytometry analysis was performed using a FACSCalibur cytometer (Becton-Dickinson, San Jose).

### Cytokine Measurement by ELISA

For cytokine measurements, culture supernatant from *Brucella*-infected BMDCs were collected after 24h of incubation and stored at −80°C. The concentrations of TNF-α, IFN-γ, IL-6, and IL-12/IL23p40 were measured using an indirect sandwich enzyme-linked immunosorbent assays (ELISA) (BD Pharmingen).

### Antigen Presentation to Naïve T Lymphocytes

A Pan T cell Isolation Kit II (Miltenyi Biotec, CA, USA) was used to isolate naïve CD4^+^ T cells and CD8^+^ T cells from uninfected BALB/c mice as described by the manufacturer. Naïve CD4^+^ and CD8^+^ T cells (at a concentration of 2×10^7^ cells/ml) were stained intracellularly at 37 °C in RPMI medium containing 1 µM carboxyfluorescein diacetate succinimidyl ester (CFSE) (Sigma-Aldrich). CFSE was quenched at room temperature for one minute with one volume of fetal bovine serum (FBS). The processed cells were then washed twice. Loaded T cells were cultured in complete RPMI medium at a concentration of 1×10^5^ cells/well in 96-well U-bottomed plates. BMDCs infected with *Brucella* for 24 h were added in a series of concentrations such that the BMDC/T – cell ratio ranged from 0.003125 to one. After five days, the cells were stained with rat anti-mouse CD4 or rat anti-mouse CD8 antibody (BD Pharmingen). The stained cells were resuspended in 2% paraformaldehyde (PFA) and stored at 4°C for analysis. A FACSCalibur cytometer was used to detect the decrease in CFSE fluorescence intensity that resulted from cellular division by flow cytometry.

### Statistical Analysis

The Student’s *t*-test (equal sample sizes, equal variance) was applied to determine statistical differences using Microsoft Excel software.
